# County-level air quality and the prevalence of diagnosed chronic kidney disease in the US Medicare population

**DOI:** 10.1371/journal.pone.0200612

**Published:** 2018-07-31

**Authors:** Jennifer Bragg-Gresham, Hal Morgenstern, William McClellan, Sharon Saydah, Meda Pavkov, Desmond Williams, Neil Powe, Delphine Tuot, Raymond Hsu, Rajiv Saran, for the Centers for Disease Control and Prevention CKD Surveillance System

**Affiliations:** 1 Department of Internal Medicine—Nephrology, University of Michigan, Ann Arbor, MI, United States of America; 2 Department of Epidemiology, School of Public Health, University of Michigan, Ann Arbor, MI, United States of America; 3 Department of Environmental Health Sciences, School of Public Health, University of Michigan, Ann Arbor, MI, United States of America; 4 Department of Urology, Medical School, University of Michigan, Ann Arbor, MI, United States of America; 5 Department of Epidemiology, Rollins School of Public Health, Emory University, Atlanta, GA, United States of America; 6 Centers for Disease Control and Prevention, Atlanta, GA, United States of America; 7 Department of Medicine University of California, San Francisco, CA, United States of America; 8 Priscilla Chan and Mark Zuckerberg San Francisco General Hospital, San Francisco, CA, United States of America; University Medical Center Groningan and University of Groningan, NETHERLANDS

## Abstract

**Background:**

Considerable geographic variation exists in the prevalence of chronic kidney disease across the United States. While some of this variability can be explained by differences in patient-level risk factors, substantial variability still exists. We hypothesize this may be due to understudied environmental exposures such as air pollution.

**Methods:**

Using data on 1.1 million persons from the 2010 5% Medicare sample and Environmental Protection Agency air-quality measures, we examined the association between county-level particulate matter ≤2.5 μm (PM_2.5_) and the prevalence of diagnosed CKD, based on claims. Modified Poisson regression was used to estimate associations (prevalence ratios [PR]) between county PM_2.5_ concentration and individual-level diagnosis of CKD, adjusting for age, sex, race/ethnicity, hypertension, diabetes, and urban/rural status.

**Results:**

Prevalence of diagnosed CKD ranged from 0% to 60% by county (median = 16%). As a continuous variable, PM_2.5_ concentration shows adjusted PR of diagnosed CKD = 1.03 (95% CI: 1.02–1.05; *p*<0.001) for an increase of 4 μg/m^3^ in PM_2.5_. Investigation by quartiles shows an elevated prevalence of diagnosed CKD for mean PM_2.5_ levels ≥14 μg/m^3^ (highest quartile: PR = 1.05, 95% CI: 1.03–1.07), which is consistent with current ambient air quality standard of 12 μg/m^3^, but much lower than the level typically considered healthy for sensitive groups (~40 μg/m^3^).

**Conclusion:**

A positive association was observed between county-level PM_2.5_ concentration and diagnosed CKD. The reliance on CKD diagnostic codes likely identified associations with the most severe CKD cases. These results can be strengthened by exploring laboratory-based diagnosis of CKD, individual measures of exposure to multiple pollutants, and more control of confounding.

## Introduction

The body of evidence suggesting that long-term exposure to air particles less than 2.5 micrometer in diameter, called fine particulate matter (PM_2.5_) air pollution, contributes to adverse health outcomes continues to grow. Early work focused on acute exposure to high levels of micro-particle air pollution where it was found to increase overall daily mortality by 7% per 50 pg/m^3^ increase in PM_2.5_, and cause-specific mortality by 25%, 11%, and 0.4% for respiratory, cardiovascular, and other causes, respectively [1]. Recently, there has been a growing interest in exploring outcomes from long-term air pollution exposure in high-risk groups, such as those with underlying cardiovascular and metabolic or respiratory disorders [2]. Even more recently, studies examining the possible effects of air pollution on the risk of chronic kidney disease (CKD) have been conducted [3–7].

Some of the first evidence of the association between PM_2.5_ and kidney disease was discovered in an ecological study of health outcomes in coal mining areas of Appalachia, where they found a 19% higher relative risk of CKD among men and a 13% higher relative risk among women in mining counties with population > 4 million compared to non-mining counties [3]. Two studies focused on the Boston community and examined estimated glomerular filtration rates (eGFR), a measure of kidney function. The first examined eGFR in patients hospitalized for acute ischemic stroke and found that individuals living closer to major roadways (<50 m) had eGFR on average lower by 3.9 ml/min/1.73m^2^ compared to patients living ≥1000 m from a major roadway [4]. The second study was a small longitudinal sample of elderly veterans and showed that individuals exposed to higher levels of ambient air pollution also had lower average estimated glomerular filtration rates and a larger annual decrease in kidney function [5]. The largest study to date have been conducted using data from the Department of Veteran Affairs where they found higher risks of multiple measures of kidney function of 20% or higher for every 10 μg/m^3^ higher PM_2.5_ level and higher rates for specific components including NO_2_ and CO [6,7].

CKD is a common condition with important long-term health implications that often goes unrecognized until advanced stages or kidney failure [8–10]. CKD currently afflicts about 27 million Americans and significantly elevates the risk of death, cardiovascular disease, end-stage renal disease (ESRD), and other complications [11]. Individuals with CKD are at an 8-10-fold increased risk of cardiovascular mortality, compared to those without kidney dysfunction [12]. CKD is typically a progressive disease with loss of kidney function over time. The rate of function loss is variable and dependent on both treatment and patient factors, including level of proteinuria, older age, diabetes mellitus, blood pressure control, obesity, metabolic syndrome, and family history of kidney disease. Early recognition and treatment of CKD and of the risk factors for CKD may slow progression of the disease [13–15]. Although much attention has been given to treatment of personal CKD risk factors, less has been focused on potential environmental contributors to the development and progression of CKD, despite the higher prevalence of both CKD and air-pollutions exposure among disadvantaged and minority populations in the United States [12].

Sources of PM_2.5_ include all types of combustion activities, such as motor vehicle emissions, power plants, and wood burning, as well as common indoor activities, such as smoking, cooking, burning candles or oil lamps, and operating fireplaces and fuel-burning space heaters (e.g., kerosene heaters) [16]. The major components of PM_2.5_ include: ammonium sulfate, ammonium nitrate, organic carbonaceous mass, elemental carbon, and crustal material [16]. Air pollution from these sources can be mitigated; thus, it is important to study its link with CKD.

Several pathophysiologic mechanisms have been proposed to explain the possible causal link between air pollution and adverse cardio-metabolic and respiratory outcomes. Many of these mechanisms are similar to factors known to play a role in initiation and progression of CKD, including: increased sympathetic nervous system activity, activation of the renin-angiotensin-aldosterone system (RAAS), vascular endothelial dysfunction, oxidative stress, inflammation, platelet adhesion and aggregation, insulin resistance, and metabolic dysregulation [17–19]. For example, there is evidence that individuals in areas with high PM_2.5_ have high levels of sympathetic activity and RAAS activation [20]. These are known contributors to initiation and progression of CKD, and treatment of individuals with medications that inhibit RAAS have been shown to slow CKD progression. Studies using experimental mouse models have also demonstrated that air pollution is associated with high mouse levels of oxidative stress and vascular endothelial dysfunction [19]. Experimental studies suggest that treating these conditions can slow CKD progression [20]. Additionally, air pollution is known to contain heavy metals. Lead, mercury, and cadmium are common heavy metal toxins known to have toxicological kidney effects at high levels. Exposure to some of these metals from the air, even at low levels, could also potentially play a role in CKD progression [21,22].

We postulate that, similar to high-risk individuals with cardiopulmonary disease, individuals with CKD would be particularly susceptible to the effects of air pollution. We, therefore, conducted an exploratory study to determine whether an association exists between county levels of ambient air pollution and CKD prevalence, controlling for potential confounders, among older adults living in the United States. Evidence of a link between air pollution and kidney disease in this study would support future studies involving individual exposures measures.

## Materials and methods

### Study sample

This study is an analysis of anonymous, secondary data sources and met University of Michigan’s Institutional Review Board standards for “Not Regulated” Status. We conducted a cross-sectional study of 1,164,057 adults ≥65 years old enrolled in the U.S. Medicare program in 2010 (Medicare 5% sample). To be included, patients were required to be enrolled in Medicare parts A and B for the full year, with no health management organization (HMO) coverage. CKD was defined using a large set of ICD-9-CM diagnosis codes indicating CKD, which are identical to the codes utilized by the United States Renal Data System [23–24]. The full set of ICD-9-CM codes were included in this study to capture all possible mechanisms for an association between PM_2.5_ and kidney disease. ICD-9-CM codes were also employed to calculate indicators of diabetes and hypertension status and were derived from both inpatient and/or outpatient diagnosis claims. This year of Medicare data was chosen to specifically align with the county-level exposure data.

### Other measures

County-level concentrations of PM_2.5_ were obtained for the year 2006 from the Centers for Disease Control and Prevention (CDC) Wonder database [25]. A full description of this data can be found on the website. Briefly, this database includes PM_2.5_ concentrations measured daily in the outdoor air and geographic aggregates of these measures of fine particulate matter. To create these data, two sources of environmental data were used as input to the surfacing algorithm, US EPA AQS PM_2.5_ in-situ data and NASA MODIS aerosol optical depth remotely sensed data and continuous spatial surfaces (grids) of daily PM_2.5_ for the whole conterminous U.S. were created for 2003–2011. County-level data were aggregated from 10 kilometer square spatial resolution grids [26]. Aggregated county-level PM_2.5_ values provided directly from the Wonder database were employed for this study for the year 2006. Particles with aerodynamic diameter < 2.5 micrometers (PM_2.5_) were the focus of this work, as evidence already exists for the effect of larger particular matter in the etiology of kidney disease and it is believed that finer particles pose a greater health risk because they are more readily inhaled and can lodge deeply into the lungs and enter the blood stream [27,28].

A 6-category ordinal variable for urban/rural status was used to account for other unmeasured differences between counties, as this measure is known to be associated with potential confounders, such as obesity, physical activity, nutrition, and poverty, as well as air pollution levels [29–33]. Data were derived from the CDC’s Urban-Rural Classification Scheme for Counties, for 2006 [34]. The six categories included: two large metropolitan groups, consisting of > 1 million residents, divided by designation as central or fringe/suburban; medium metropolitan with 250,000–999,999 residents; small metropolitan with < 250,000 residents; and two non-metropolitan categories, micropolitan if containing an urban cluster of > 10,000 residents and non-core if no urban cluster. County-level data on poverty and education, from the 2006 Behavioral Risk Factor Surveillance System BRFSS Supplement [35], were examined as markers of socioeconomic status, but were not associated with CKD in our analysis after accounting for the urban-rural status of each county and were therefore not used in the final models.

### Statistical analysis

Although the main exposure variables in this analysis are ecologic (aggregated) measures of PM_2.5_ at the county level, the unit of analysis is the individual level outcome of CKD status and all covariates except urban/rural status are measured at the individual level [36]. The county of residence for every individual in the study population was indicated by the 5-digit Federal Information Processing Standard (FIPS) codes and was used to merge the air pollution data to each patient in the sample [37].

Descriptive statistics are presented for the total sample and the sample stratified by the median PM_2.5_ concentration (12.2 μg/m^3^), which lies very near the middle of the bimodal distribution of this measure. The individual-level diagnosis of CKD was modelled as the outcome, using modified Poisson regression with robust errors. This modeling approach was chosen, as opposed to logistic regression, because it yields estimates of prevalence ratios (PRs), rather than odds ratios [38,39]. The final model accounted for clustering of the outcome within counties, using a compound symmetry covariance matrix. Two parameterizations of county-level mean PM_2.5_ were examined: as a continuous variable (expressed for an increase of 4 μg/m^3^, which is approximately the interquartile range) and by quartiles. All PM_2.5_ measures are reported in micrograms per cubic meter (μg/m^3^). PR estimates, comparing mean exposure levels, were adjusted for the following available potential confounders: age, sex, race/ethnicity (Non-Hispanic White, Non-Hispanic Black, Hispanic, Asian, North American Native, Other, and Unknown), diagnosed hypertension, diagnosed diabetes, and urban/rural status.

## Results

**Fig 1:**
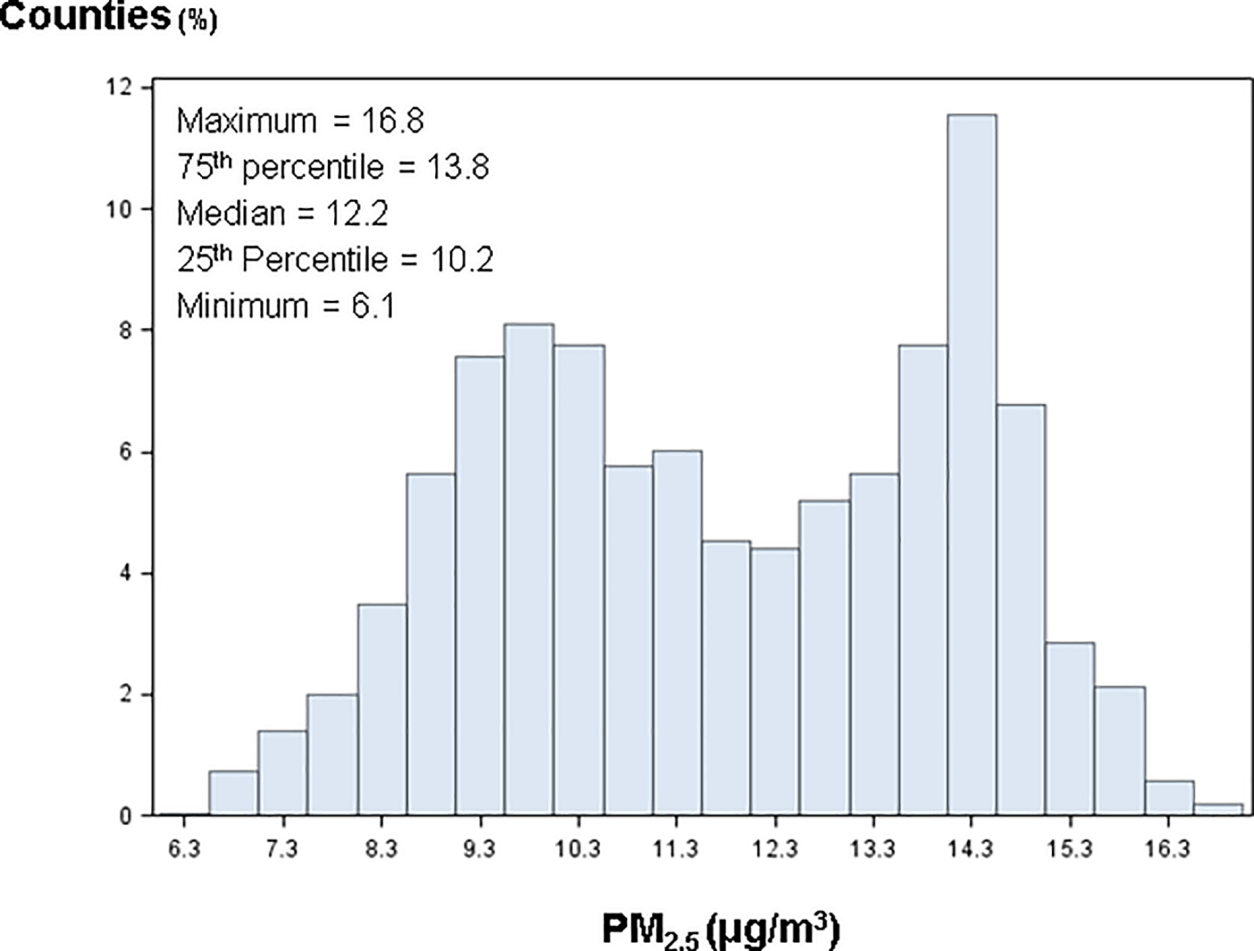
Histogram of county-level PM_2.5_. Data Source: http://wonder.cdc.gov/nasa-pm.html.

Of 3,143 U.S. counties, CKD diagnosis information was available for enrollees within 3,108, PM_2.5_ data was available for 3,111, and both variables were available for 3,049 counties. The overall prevalence of diagnosed CKD in the sample was 17.2%. When examined at the county-level, the median county-level prevalence of diagnosed CKD in the Medicare population was 16%, ranging from 0%-60%, with an interquartile range of 13%-19%. The median county-level PM_2.5_ concentration was 12.2 μg/m^3^, ranging from 6.1 to 16.8 μg/m^3^, with an interquartile range of 10.2–13.8 μg/m^3^. The distribution of county-level PM_2.5_ concentration was bimodal, as displayed in Fig 1.

When examining characteristics of Medicare enrollees by the two clusters of PM_2.5_ concentration: high (PM_2.5_ > 12.2 μg/m^3^) and low (PM_2.5_ ≤ 12.2 μg/m^3^), we see that enrollees in counties with higher PM_2.5_ were slightly younger, contained a higher proportion of females and non-Hispanic Blacks, higher prevalence of both diabetes and hypertension, and a higher proportion of enrollees living in large metropolitan areas (Table 1).

**Table 1:** Characteristics of the Medicare enrollees stratified by level of exposure to county-level PM_2.5_ (μg/m^3^)^ᵼ^.

Measure	Low PM_2.5_ (≤ 12.2 μg/m^3^)	High PM_2.5_ (> 12.2 μg/m^3^)	
Age (years, SD)	75.4 (7.7)	75.2 (7.6)	
Male (%)	42.1	40.6	
Race/Ethnicity: (%)			
Non-Hispanic White	90.2	86.0	
Non-Hispanic Black	3.6	11.0	
Hispanic	1.9	0.8	
Other/Missing	4.3	2.2	
Diabetes* (%)	28.6	32.2	
Hypertension* (%)	73.2	77.4	
Rural Urban Status:			
Large Metro	39.8	47.3	
Small-Medium Metro	34.0	30.2	
Micropolitan or non-core	26.2	22.5	

*Diabetes and Hypertension identified by ICD-9-CM codes

^ᵼ^ All p-value<0.0001 when comparing measures between low and high PM_2.5_ counties.

**Fig 2:**
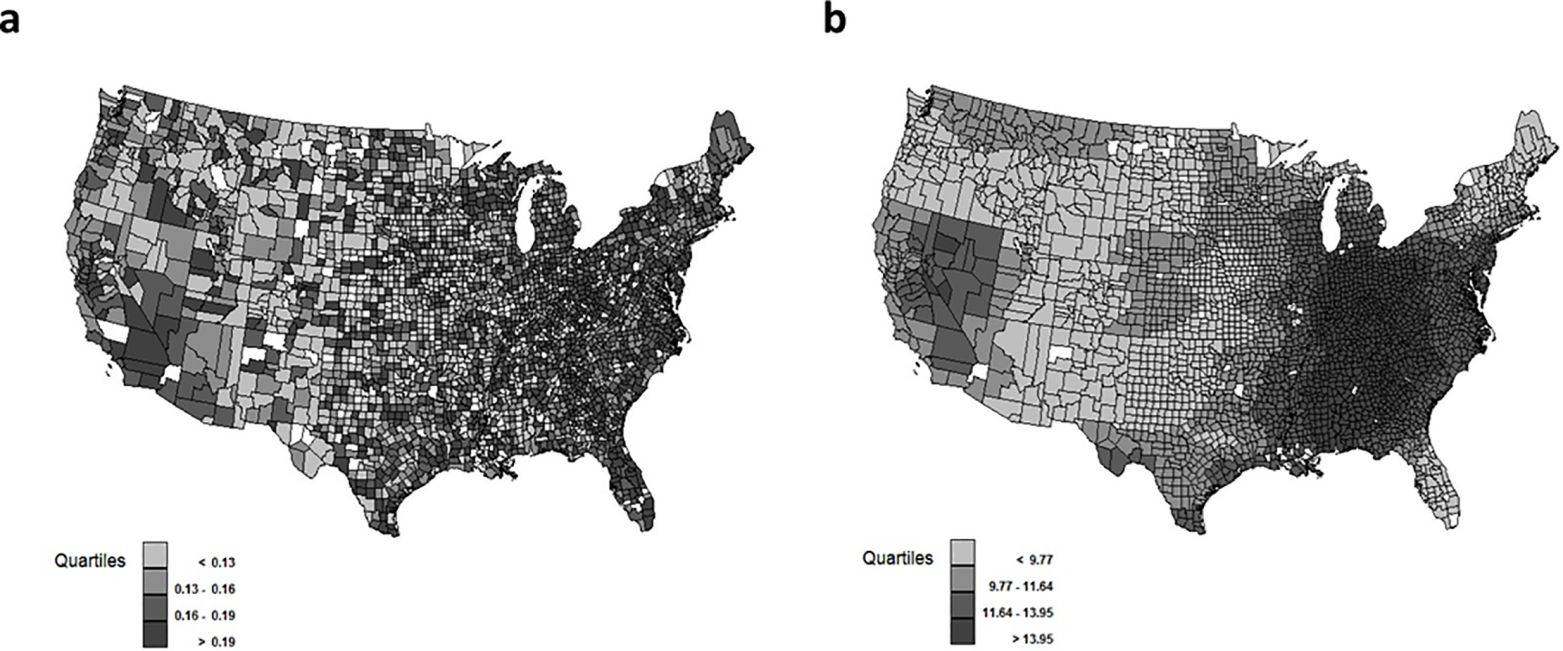
U.S. county distributions. a: Proportion of Medicare Sample with Diagnosed CKD by County, b: Average PM_2.5_ (μg/m3) by County, Non-shaded counties had missing information and were not used in the analysis.

Maps of the county-level quartiles of both diagnosed CKD and mean PM_2.5_ concentration are displayed in Fig 2A and 2B. No striking patterns of diagnosed CKD appear, though lower prevalence are observed between Montana and New Mexico and West Texas. Fig 2B illustrates higher concentrations of PM_2.5_ from the Ohio Valley southward along the Mississippi, in Nevada and eastern California, and the Appalachian mountains.

There was a clear pattern of higher prevalence of diagnosed CKD in large central metropolitan areas (18.4%), decreasing steadily to 16.0% and 15.1% in micropolitan and non-core counties, respectively (p<0.0001). Due to this observed association, all models examining the association between diagnosed CKD and fine particulate matter in air accounted for the county’s urban-rural status, as well as the risk factors shown in Table 1.

**Fig 3:**
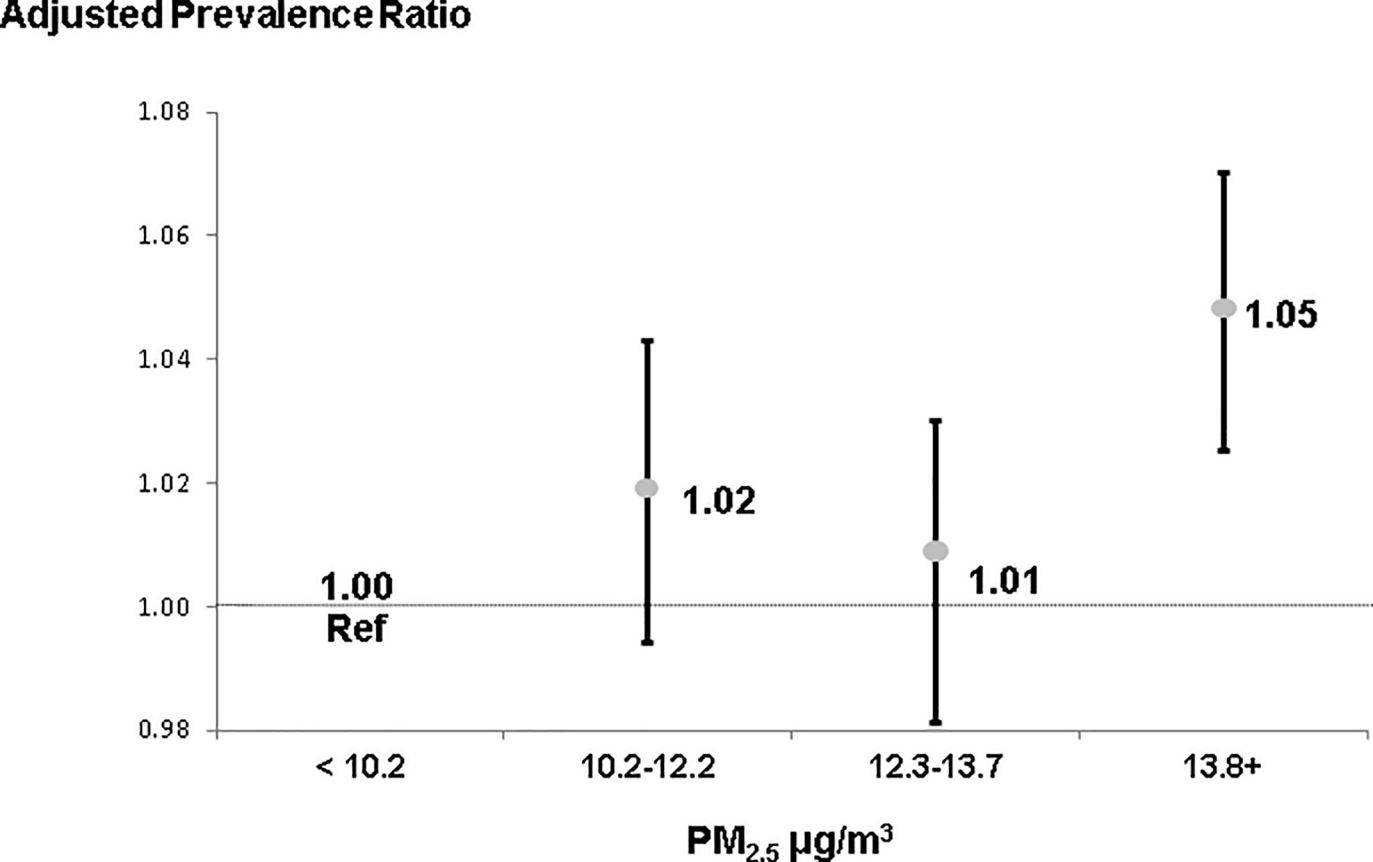
Prevalence ratios for CKD by county-level PM_2.5_*. Adjusted models included age, sex, race, diabetes, hypertension, and six categories of county urban/rural status. NOTE: Vertical bars display the 95% Confidence Intervals of the estimates.

We examined PM_2.5_ concentration as both a continuous and as a 4-category ordinal variable (quartiles) in separate analyses. In unadjusted models, a 4 μg/m^3^ higher PM_2.5_ concentration was associated with a 1.12 (95% CI: 1.10–1.14) PR of diagnosed CKD. After adjustment for patient characteristics and urban/rural status, the PR was 1.03 (95% CI: 1.02–1.05). Categorizing average PM_2.5_ level in quartiles and treating the first (lowest) quartile as the reference group, where PM_2.5_ <10.2 μg/m^3^, the adjusted PR was 1.02 (95% CI: 0.99–1.04) for counties in the second quartile, 1.01 (95% CI: 0.98–1.03) for the third quartile, and 1.05 (95% CI: 1.03–1.07) for the fourth quartile where the average PM_2.5_ level was ≥13.8 μg/m^3^ (Fig 3).

## Discussion

In a large population of subjects, aged 65 years and older, enrolled in the Medicare insurance program of the United States, county-level concentration of ambient PM_2.5_ was positively associated with diagnosed CKD. This association was attenuated, but remained statistically significant even after adjusting for individual demographic characteristics, diagnosed hypertension and diabetes, and county level urban-rural status. In all models, higher average concentrations of PM_2.5_ were associated with higher prevalence of CKD. We also found no evidence that this association is due to differences in the age, sex, race, diabetes, or hypertension prevalence differences between regions. Although there could be other confounders affecting this relationship, these characteristics are some of the most common risk factors related to CKD. While the effect size of PR = 1.05 may not seem large, one should remember that this effect is for all residents of the county, not just those of a specific age, race/ethnicity, or with a certain comorbid condition. The effect size is also similar to those found in studies of other chronic disease outcomes [40].

This finding is important in regards to standards for air quality. The U.S. Environmental Protection Agency currently sets the lower limit threshold for PM_2.5_ at 12 μg/m^3^; which could be interpreted to mean that levels lower than this threshold are deemed safe, and *vice versa* [27]. This value is much lower than the daily level typically considered healthy for sensitive groups (~40 μg/m^3^) and almost half of the counties had mean PM_2.5_ levels that were above these guidelines [19]. Moreover, it is not entirely clear that lower levels are indeed safe for those with health conditions that raise their risk of cardiopulmonary complications. If these findings can be validated in future research, they may point to the importance of assuring adequate protection from environmental air pollution for individuals at risk of, or already suffering from, varying severity of CKD.

The findings from this study are consistent with results from studies that have examined the association between air pollution and other chronic conditions, such as cardiovascular and pulmonary disease, but these studies are few in number. In one study, Schwartz, *et al*.[40], found that after controlling for age, race, sex, and cigarette smoking, annual average total suspended particulate concentrations were associated with increased risk of chronic bronchitis (odds ratio = 1.07; 95% CI: 1.02–1.12). Most studies of air pollution and its effects on health have been limited to looking at cardiac or mortality events [41–45]. Our results are also consistent and extend recent work examining associations between air pollution and kidney disease [3–7], while focusing on a large, novel population of elderly Medicare recipients. Future research among kidney disease patients will examine hospitalization and mortality, as well as incidence of kidney disease in this patient population.

The significant overlap in risk factors, pathogenesis, progression, and complications of cardiovascular and kidney disease is, in general, well recognized [46,47]. The cardiovascular system is especially vulnerable even in early stages of CKD with early onset of endothelial dysfunction [48]. Free radical-mediated injury, activation of vasoactive and pro-inflammatory cytokines, the central role of activation of renin-angiotensin-aldosterone, abnormal autonomic imbalance with abnormalities in heart rate variability, increased arterial stiffness, accelerated atherosclerosis, and a high propensity to acute cardiovascular events including sudden death are common to both cardiovascular disease (CVD) and CKD [49–51]. A number of other metabolic abnormalities unique to the uremic milieu additionally render patients with CKD even more vulnerable to environmental and other insults/stressors, such as air pollution. The kidney, while seemingly remote from air in the environment, is intimately linked to the circulatory system–by virtue of the high rates of blood flow through its parenchyma–and therefore to the environment, thereby sharing vulnerability with the respiratory and cardiovascular systems [52].

We recognize that the association between air pollution levels and prevalence of diagnosed CKD does not indicate a (causal) effect and may be confounded by county-level differences in a number of unmeasured characteristics, including health system capacity and other environmental factors. By adjusting for each county’s urban-rural status, we have aimed to minimize this potential confounding. This study was restricted to a population at the highest risk for kidney disease, Medicare enrollees (aged 65 years and older), and the results are not generalizable to younger age groups. While older individuals are at high risk, an examination of younger ages would benefit any future work. The main methodological limitations of the current work are its cross-sectional design and lack of individual-level exposure data.

This study was also limited to the use of administrative healthcare claims data for identification of CKD. It is likely that individuals with early stages of CKD do not have a diagnosis and are therefore classified as non-cases. We chose to use the list of ICD-9 codes utilized by the United States Renal Data System, which includes all diagnoses of CKD, because although some diagnoses, such as posterior urethral valves or pyelonephritis, are not likely associated with air pollution, we cannot exclude this possibility based on our study. Also, a systematic review of coding for CKD and related conditions has shown the sensitivity of using only diagnostic codes to be low, typically under 50% [53]. Moreover, these conditions would be extremely rare in the population under study. The reliance on claims data also excluded an examination of these associations by stage of CKD. Future work would benefit by focusing on cohorts that include laboratory data for use in classifying individuals into appropriate CKD categories. The authors also acknowledge that there may be air pollution data quality limitations and refer the reader to the CDC Wonder website for details.

If indeed a variety of studies consistently further support the hypothesis that air pollution is a risk factor for kidney disease incidence, progression and other complications, it may lend greater impetus to encourage public health and clinical efforts to not only offer greater protection to these higher risk individuals, but also to establish evidence at lower thresholds for air pollution standards, in general. Specific toxins in the environment (e.g., lead, aristolochic acid, heavy metals, etc.) have definitively been linked with nephrotoxicity, and minimal exposure has been advised. It is well known that patients with kidney disease are especially susceptible to cardiopulmonary complications and when in highly polluted areas, may benefit from the use of preventive measure that are relatively simple and easy to implement. It may also be advisable for such individuals to consider limiting long hours commuting to work in high traffic areas where there is significantly higher exposure to environmental pollutants and other stressors [54–55].

Although this study included over one million individuals, the cross-sectional design and lack of individual exposure data severely limit causal inference. It does, however, support further research in this area, using more detailed air pollution exposure data mapped to the patient- or zip-code level rather than the more crude averaged, county-level estimates utilized in this study. If this association is borne out by future studies, it would have clinical and public health implications for reducing air pollution exposure for those with CKD and also for those at risk for the condition. The potential public health significance of this finding is even greater for regions and countries with much higher levels of air pollution than the United States.
